# A Novel PCR-Based Tool to Trace Oenological *Saccharomyces cerevisiae* Yeast by Monitoring Strain-Specific Nucleotide Polymorphisms

**DOI:** 10.3390/foods14132379

**Published:** 2025-07-04

**Authors:** Anna Baldisseri, Davide Santinello, Sara Granuzzo, Martina Frizzarin, Fabio De Pascale, Geppo Sartori, Paolo Antoniali, Stefano Campanaro, Raffaele Lopreiato

**Affiliations:** 1 Department of Biomedical Sciences, University of Padova, Via U. Bassi 58, 35131 Padova, Veneto, Italy; anna.baldisseri@gmail.com (A.B.); sara.granuzzo@studenti.unipd.it (S.G.); geppo.sartori@unipd.it (G.S.); 2 Department of Biology, University of Padova, Via G. Colombo 3, 35131 Padova, Veneto, Italy; davide.santinello.1@phd.unipd.it (D.S.); fabio.depascale@unipd.it (F.D.P.); stefano.campanaro@unipd.it (S.C.); 3 Italiana Biotecnologie Srl, Via San Cassiano 99, 28069 Trecate, Novara, Italy; martina.frizzarin@italianabiotecnologie.it (M.F.); paolo.antoniali@italianabiotecnologie.it (P.A.)

**Keywords:** *Saccharomyces cerevisiae*, molecular genotyping, yeast strains traceability

## Abstract

*Saccharomyces cerevisiae* plays a fundamental role in winemaking, not only driving alcoholic fermentation but also producing secondary metabolites that contribute to the organoleptic properties of wine. To ensure consistent quality and process efficiency, wineries commonly employ selected starter strains. Accordingly, the ability to control strain purity and traceability is of critical importance. Currently, the inter-delta PCR method is widely used for the strain-specific genotyping of *S. cerevisiae*. However, its resolution diminishes when analyzing genetically similar strains, such as those isolated from related grape types or during genotyping of large yeast collections. To address this limitation, we developed a novel strategy that integrates computational and experimental approaches to identify highly specific allelic variants (single nucleotide polymorphisms, SNPs) within the *S. cerevisiae* genome. Comparative genomic analysis of twenty-eight different strains led to the identification of multiple strain-specific SNPs. From these, nine SNPs spanning five strains were selected and validated through targeted PCR assays. These assays confirmed the feasibility of using SNPs as reliable genetic markers for strain discrimination and traceability. Overall, our findings demonstrate that this SNP-based approach, implemented via multiplex allele-specific (AS) PCR assays, offers a rapid, cost-effective, and highly discriminatory alternative to current genotyping methods, particularly for differentiating closely related strains.

## 1. Introduction

Wine production is a multi-step process in which fermentation plays a crucial role by converting the sugars in the must into alcohol and carbon dioxide (CO_2_). It is driven by specific microorganisms, naturally found on grape skins, which are well-adapted to the environmental conditions present when the grapes are crushed (e.g., high sugar concentration). Among these microorganisms, yeasts of the *Saccharomyces cerevisiae* species are predominant because of their strong fermentative metabolic capabilities and their high resistance to alcohol and various chemical/physical stresses (e.g., temperature, pH, metals). These conditions often inhibit other microorganisms, such as bacteria and other fungi, which share the same ecological niche [1,2].

Fermentation of the must can occur spontaneously by relying on the yeast cells present on the grapes, though this may lead to significant variations in the organoleptic and aromatic qualities of the final product. Over the years, various *S. cerevisiae* strains have been isolated and selected for different styles of winemaking [3,4]. Today, these strains are widely used as yeast starters, particularly in industrial wineries, due to their ability to ensure efficient fermentation and to contribute to the aromatic characteristics that define specific types of wine [5]. Indeed, during fermentation, yeast not only produces ethanol but also generates aromatic compounds, such as esters, ketones, and higher alcohols [6], which influence the wine’s bouquet. Over four hundred such compounds are produced by yeast cells, and the type and concentration of these end-products are determined by the specific strains involved in fermentation [7]. Different strains impart distinct flavors to the same wine, depending on their unique genetic traits, which are reflected in their metabolic capacities [8].

The ongoing isolation of *S. cerevisiae* strains with peculiar phenotypes and, subsequently, their employment in industrial winemaking, further require specific experimental techniques to perform the quality control of commercial products. These strains are normally provided as active dry yeast (ADY) in order to ensure the desired standards, as well to allow product traceability. Generally, yeast identification and classification rely on phenotypic, morphological, and physiological characteristics, which are labor-intensive and prone to inaccuracies [9]. Concerted efforts so far resulted in the development of rapid and unequivocal methods for identifying yeasts at both species and strain levels [10]. In this context, DNA-based techniques are mostly used because they are independent of gene expression and therefore unaffected by observable phenotypic characters. Multiple procedures for yeast genotyping have been established so far. To identify yeast species, the PCR amplification of ribosomal genes is often employed, followed by the restriction of the amplified products and the analysis of DNA fragment length by agarose gel electrophoresis (PCR and Restriction Fragment Length Polymorphism, PCR-RFLP) [11,12]. Although some restriction patterns have been reported for oenological yeasts, the strains not included in such databases cannot be identified, limiting the use of the technique to discriminate newly isolated or genetically similar strains [13,14]. Alternatively, PCR-derived rDNA gene amplicons can be discriminated in a sequence-specific manner, by either denaturing-gradient (DGGE), temperature-gradient (TGGE), or polyacrylamide gel electrophoresis [15,16,17]. Additionally, ribosomal DNA can be also investigated by Sanger sequencing the D1/D2 region of the 26S gene, leading to yeast species identification through the comparison with data already available [10]. Finally, multiple yeast species can be simultaneously analyzed during real-time PCR experiments, allowing the detection and quantification of a species even at low concentrations, but such an approach may become expensive, requiring not only specific instruments and reagents (e.g., fluorescent probes), but also trained personnel to set up and perform the experimental assays [16]. Strain-level yeast identification is commonly based on genome fingerprinting, producing a peculiar pattern through the digestion or amplification of specific DNA regions, which allows for the comparison of different strains [16]. The restriction analysis of mitochondrial DNA (mt-RFLP) can efficiently identify the *S. cerevisiae* strains, as yeast mtDNA is endowed with high variability [18]. Genetic polymorphisms are among the best targets to identify yeast strains through PCR-based amplification methods. Genomic DNA subjected to the “Randomly Amplified Polymorphic DNA” (RAPD) PCR analysis produces strain-specific amplified fragments of varying sizes, resulting in a pattern that can be compared to other reference strains [16]. Additional polymorphic loci of the yeast genome can be further investigated by specific PCR analysis, as microsatellites [19], or Intron Splice Sites (ISSs) [10]. Moreover, the Amplified Fragment Length Polymorphism (AFLP) technique is also used to identify yeast strains, which is performed by digesting the genomic DNA, attaching adaptors to the fragments, and amplifying them through PCR [16]. However, the use of these methods may be limited by either the low reproducibility of the experiments (as minimal variations in DNA isolation and/or manipulation can affect the outcome), or special requirements of time and materials, often including peculiar instruments.

Given its versatility, today the most used PCR-based technique is inter-delta analysis, where randomly distributed genomic “delta” regions are amplified using two specific primers [20]. Many commercial *S. cerevisiae* strains have been successfully discriminated by this method, unveiling distinct amplicon patterns for different strains [10]. Among the PCR-based assays, inter-delta analysis has proven to be the most discriminating (and cost-effective) among various fingerprinting methods [21]. However, very similar yeast strains cannot be discriminated by inter-delta analysis, as they may produce identical patterns despite having distinct phenotypic characteristics [20,22]. Recently [23], it has been reported that the sequencing of DNA fragments derived from inter-delta amplification can further lead to differentiate strains with identical patterns. In a commercial setting, however, it could be too expensive to use multiple sequencing experiments routinely in systematic quality control assays, also requiring a longer processing time.

In this study, we aimed to overcome the limitations of inter-delta analysis for the identification of genetically similar *S. cerevisiae* strains, while maintaining the efficiency, speed, and low cost of simple PCR-based approaches. Our method requires the availability of NGS data of the strains of interest, displaying identical inter-delta profiles, in order to identify the single nucleotide polymorphisms (SNPs) that can distinguish them. The genetic differences found by computational analysis allow us to design specific primers for the unique identification of the SNPs, and consequently, of the strain. This approach enables the continued use of a rapid and inexpensive PCR-based method for the screening or quality control of the strain(s) of interest, upon genome sequencing.

## 2. Materials and Methods

### 2.1. Yeast Manipulation and Genomic DNA Extraction

The *S. cerevisiae* strains analyzed in this study belong to the private collection of Italiana Biotecnologie Srl (Trecate, Novara, Italy). Additional information on some strains (e.g., origin, source, applications) are reported in the Supplementary Materials. Common laboratory strains (W303, S288c) were also used as references. Yeast cells were maintained and cultured at 28 °C in standard complete medium (YPD) (yeast extract: 10 g/L, Bacto Peptone: 10 g/L, glucose: 20 g/L), or in WL Nutrient Agar, commonly employed for the growth of yeasts for oenological or brewing use, as reported in [24,25]. Genomic DNA extraction from yeast cells was performed by using the ZYMO RESEARCH (Quick-DNA Fungal/Bacterial Kit, D6005, Irvine, CA, USA) columns, following the manufacturer’s instructions; 1 μL of purified DNA (20 ng) was used as a template for the PCR reactions. DNA quality was assessed by NanoDrop (Thermo Fisher Scientific, Waltham, MA, USA) and agarose gel electrophoresis. Media components and chemicals, as reagents for auxotrophic requirements, were obtained from Difco (Thermo Fischer Scientific, Carlsbad, CA, USA) and Sigma-Aldrich (Saint Louis, MO, USA).

### 2.2. Yeast Strain Mixtures

Binary and multiple mixtures of different strains were prepared combining an equal amount of yeast cells of each strain. Briefly, the cells grew overnight in liquid YPD complete medium (5 mL) and yeast cultures were diluted to have the same cell concentration, based on optical density (final OD_600_ = 0.1). Equal volumes (0.5 mL) of each strain with diluted culture, containing an identical number of cells, were mixed and further diluted (1:10,000). A total of 100 µL of the mix was finally plated on solid YPD complete medium and incubated at 28 °C for 2–3 days. Several colonies were selected, and genomic DNA was extracted from each single colony, as mentioned.

### 2.3. Primer Design

Nine strain-specific single nucleotide polymorphisms (SNPs) were selected to analyze five yeast strains: three SNPs were uniquely identified in the C1.B strain, two SNPs in either C1.A and C2.A strains, and one single SNP in either C1.C and C1.D strains. Primers were designed with the software PRIMER v.1, freely available online (http://primer1.soton.ac.uk/primer1.html, accessed on 26 June 2025) [26] (software parameters are detailed in the Supplementary Materials). As a result, two primer pairs for a given SNP can be obtained: one specific for the wild-type allele (WT alleles: forward and reverse) and one for the SNP variant allele (AS forward and reverse primers). The primer pairs were selected depending on the size of amplification products, required to differ by (at least) 200 bp, as well as a similar melting temperature (Tm). A destabilizing mismatch was added at position -2 from the 3′- end of both the WT and SNP variant allele-specific primers to improve their specificity for the target sequence [27]. Primer design was performed on the *S. cerevisiae* strain reference W303 and further aligned with the five yeast strains’ genomes, in order to exclude possible mismatches that could invalidate the PCR reaction. Wild-type allele-specific primers were combined with SNP variant allele-specific primers to perform the preliminary set up of PCR assays, and only SNP specific primers (i.e., AS primers) were used in the subsequent experiments. The sequences of the eighteen AS specific primers successfully tested in this study are reported in Supplementary Table S1.

### 2.4. Multiplex Allele-Specific Primer PCR (AS-PCR)

The PCR assays protocol was carefully optimized to obtain the most specific pairing between DNA template and primers, combining standard and touch-down PCR techniques [28]. The final method consisted of two subsequent cycling phases: the first phase (touch-down) started with an annealing temperature of 69.5 °C, which gradually decreased by 1 °C per cycle until it reached 61.5 °C (9 cycles in total). Then, in the second (standard) phase, the annealing temperature was maintained for 25 cycles. The entire PCR protocol consisted of 34 cycles: 2 min at 98 °C (activation of hot-start DNA polymerase), 9 cycles of touch-down PCR (denaturation, 95 °C for 30 s; annealing temperature, 69.5 °C (decreasing by 1 °C per cycle) for 30 s; polymerization, 72 °C for 1 min), followed by 25 cycles of standard PCR (95 °C for 30 s; 61.5 °C for 30 s; 72 °C for 1 min), and ended by a final elongation step (5 min at 72 °C). PCR reactions were performed in a final volume of 30 µL containing on average 20 ng of genomic DNA as the template, 0.2 mM of dNTPs, 6 µL of 5× Reaction Buffer (with MgCl_2_), 1,5 U of Taq enzyme (GoTaq^®^ Hot Start Polymerase, Promega, Madison, WI, USA), and 0.5 µM of each primer (from two to twelve). All primer pairs were either tested individually or combined simultaneously in multiplex reactions, as indicated in each experimental assay. Primers were validated by PCR using as a template the genomic DNA of the strain carrying the target SNP, whereas the genomic DNA of either laboratory strains or yeast strains lacking the target variant was used as a negative control. Amplified DNA fragments were finally analyzed by electrophoretic separation on 1.5% agarose gel and visualized after staining with ethidium bromide.

### 2.5. Inter-Delta Assay

The inter-delta genotyping assay was performed by PCR using the delta-12 and delta-21 primers, as described in [20]. Since we used a different DNA polymerase, the original PCR protocol was slightly modified by adapting the time intervals and by the addition of 10 amplification cycles: 2 min at 95 °C (initial denaturation); 45 PCR cycles (95 °C, 30 s; 42 °C, 40 s; 72 °C, 60 s); 72 °C, 5 min (final extension). The reaction mixture (total volume: 25 µL) contained 0.5 µM of each primer, 30 ng of genomic DNA, 0.2 mM of dNTPs, 5 µL of 5× reaction buffer (with MgCl_2_), and 1.25 U of GoTaq^®^ polymerase enzyme. PCR products were visualized after electrophoretic separation on 1.5% agarose gel stained with ethidium bromide.

### 2.6. Variant Calling and Analysis

Whole genome sequencing of the strains was performed as previously described [29]. Briefly, the genomic DNA was extracted with a Quick-DNA Fungal/Bacterial Microprep Kit (Zymo Research, Irvine, CA, USA) and processed for library preparation at the sequencing facility of the CRIBI Biotechnology Center (University of Padova, Italy). A TruSeq DNA PCR-Free Library Prep Kit and Reagent kit v2 (paired-end) were used, and sequences were generated using a NextSeq 500 platform (Illumina Inc., San Diego, CA, USA).

Variant calling was performed with GATK41 software (v. 4.0.3.0) using as a reference genome *Saccharomyces cerevisiae* S288C (R64-1-1). Analysis was performed following GATK Best Practices [30], as previously described [29]. Briefly, (I) reads were aligned using bwa mem (v0.7.17) [31]; (II) to perform the base-quality score recalibration, a previously published dataset [32] was reduced in order to include only variants present in at least 6% of the strains and used as a “truth” set; (III) variant calling was performed with the haplotype caller algorithm; and (IV) variant filtering was performed using the variant quality score recalibration workflow. Three different subsets of the same reduced dataset were used to train the recalibration model.

Strain-specific variants, i.e., variants only found in one of the analyzed strains, were identified through the “bcftools view” command with the “private” option set. Variants selected for AS-PCR were located in regions with coverage higher than 50× in all the isolates.

Starting from biallelic SNPs, the distances between samples were estimated with custom Python scripts by performing pairwise comparisons in order to calculate the number of variants not shared between each comparison. The resulting matrix was used to construct a neighbor-joining tree with PHYLIP software (v.3.698) [33]. The tree was produced with Dendroscope7.

### 2.7. Yeast Competitive Assay in Stuck Fermentation Conditions

Eleven different yeast strains (C1.A-D, C2.A, C3.A, U_4_, U_10_, U_15_, U_16_, and U_17_) were used in this assay. Strains were individually grown overnight at 28 °C in liquid YPD medium, and the cell concentration was determined for each strain using a Burker chamber (Brand^TM^ Blaubrand^TM^, Sigma-Aldrich, Saint Louis, MO, USA). A mixture composed of the eleven strains was then prepared, where the yeast cell concentration was similar for each strain (4.5 × 10^5^ cells/mL). This mixture was used to inoculate two independent bottles with a final concentration of 5 × 10^6^ cells/mL. Each bottle contained 350 mL of commercial white wine (microbiologically sterile, 10.9% *v*/*v* ethanol), supplemented with an appropriate amount of fructose (25 g/L) to produce additional ethanol (approximately 1–1.5% *v*/*v*) through fermentation. The strains were not preliminarily acclimatized to increase the biotic stress during the assay. Static fermentation was carried out at 20 °C and constantly monitored by a Ankom ^RF^ Gas Production System, according to the manufacturer’s instructions (ANKOM Technology, 2052 O’Neil Road, Macedon, NY, USA). The pressure increase, due to CO_2_ released during fermentation, was recorded by the system every 30 min and used to indirectly determine the production of ethanol, finally represented as fermentation kinetics. After 22 days, when the ethanol levels stabilized (1.0–1.2% *v*/*v*), isolation plates were then prepared, spreading selected dilutions from bottles 1 and 2 on WL nutrient agar plates. Yeast manipulation, fermentation assays, and colony isolation were performed in sterility to avoid contamination. After incubation for 48 h at 28 °C, twenty-six colonies were randomly selected (thirteen from the plates of each bottle), representing 15% of the total colony number. The genomic DNA extracted from each colony was used for a first genotyping assay by inter-delta PCR; then, a multiplex AS-PCR was performed, as already described in previous sections.

## 3. Results

### 3.1. Yeast Strain Selection

In this study, we analyzed *S. cerevisiae* strains belonging to a private collection (Italiana Biotecnologie SRL, Trecate, Novara, Italy), which have been isolated by the company over the years from different geographical areas (e.g., USA, France, Italy). Over time, the strains have been characterized in the company’s laboratory for specific properties relevant to oenological purposes (fermentative aptitudes, stress resistance, release of secondary metabolites), and promising strains were further deposited and conserved in the DBVPG collection of industrial yeasts (University of Perugia, Perugia, Italy). Today, some collection’s strains are commercially produced as “starters” and used in worldwide wineries. Notably, winemaking-related phenotypes of the strains have no impact on the conclusions of the present study, but additional information on some strains has been reported in the Supplementary Materials.

**Figure 1:**
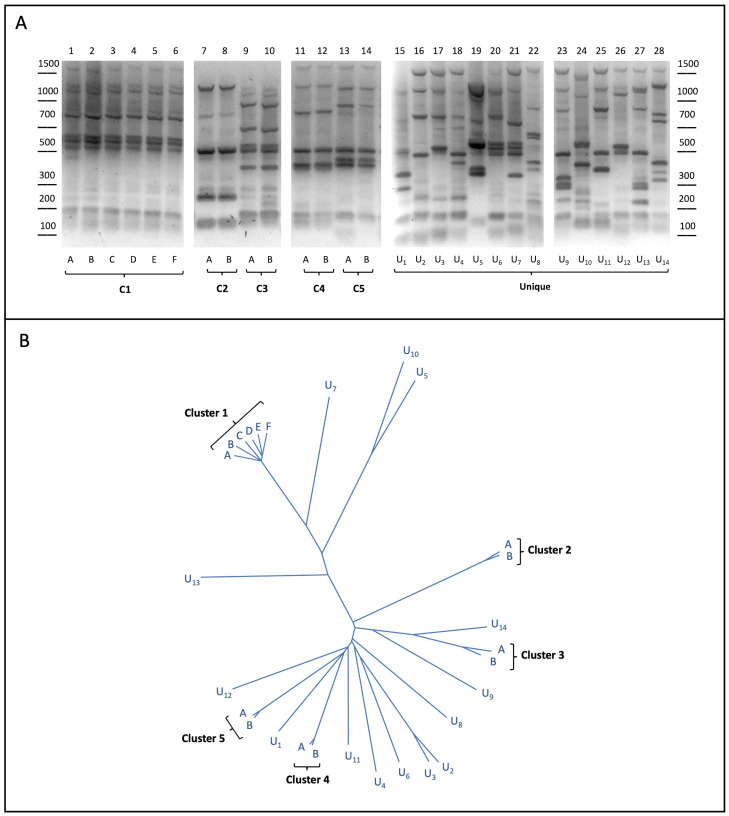
(**A**) Yeast strain genotyping. Genomic DNA purified from the twenty-eight yeast strains was used as the template in inter-delta PCR assays. DNA products were subjected to agarose gel separation and visualized upon staining. Yeast strains sharing identical profiles were grouped in multiple or binary clusters (C1–C5); (**B**) neighbor-joining tree of the twenty-eight yeast strains involved in this study. Length of the branches connecting two strains is proportional to the similarity in their SNP profiles.

A subset of twenty-eight yeast strains from the collection were analyzed by the inter-delta PCR assay, commonly used to perform *S. cerevisiae* genotyping, as already reported [10,34,35]. As shown in Figure 1A, our analysis for the twenty-eight strains unveiled nineteen different banding patterns, indicating that some strains (U1–U14) were characterized by specific (unique) inter-delta profiles, whereas common, yet different, patterns were shared by two (or more) of the other fourteen strains. The comparison between their inter-delta profiles finally resulted in the definition of five yeast clusters composed by either multiple (C1) or two (C2–C5) different strains.

Interestingly, strains belonging to the same inter-delta clusters also shared similar phenotypic traits related to winemaking, such as the fermentative power and the sensitivity to copper (Supplementary Figure S1), but they could be also endowed with strain-specific properties, as observed for the C1.A and C1.B strains, preferentially used for the fermentation of red and white grape musts, respectively (see Supplementary Materials). Such phenotypic differences could be related to strain-specific genetic features, which could not be detected by inter-delta analysis. These include minor changes in the genome, i.e., single nucleotide polymorphisms (SNPs), small insertions or deletions (InDel), repetitive loci (DNA satellite), or larger mutations, like copy-number variation events and/or translocations. As NGS genomic data were already available (in the company’s database) for all twenty-eight strains considered here, we performed the genome-wide identification of the strain-specific (unique) genetic variants, as described in the following section.

### 3.2. Computational Identification of Strain-Specific SNPs

Computational results were obtained as carefully reported in Section 2 (Materials and Methods Section). According to genomic sequencing previously performed on a large number of *S. cerevisiae* isolates [29], the number of variants compared to the reference genome identified in the twenty-eight strains considered here ranged between 54,000 and 70,000, while strain-specific variants identified were distributed as reported in Table 1. Notably, similar values have also been obtained by the analysis of yeast strains with different origins and biotechnological applications (e.g., enological, beer, sake, etc.) [32,36].

Bioinformatic analysis of the yeast sequences further resulted in the phylogenetic tree shown in Figure 1B, which reflected the similarity between their genomes. Notably, the strains with identical inter-delta profiles were closer together in the tree, forming different subgroups, which are consistent with the inter-delta clustering described above. Importantly, all the comparisons, including those performed between strains of the same cluster, revealed the presence of multiple strain-specific variants. In order to select suitable variants for our strategy, small insertions/deletions (InDels) were excluded, selecting either homo- or heterozygous SNPs, which could be more stringent as genetic markers. Once the unique variants were filtered based on the quality assessment, a list of strain-specific SNPs was generated (Table 1 and Supplementary Table S2).

Starting from the entire dataset (Supplementary Table S2), several SNPs were selected to perform the experimental allele-specific (AS) PCR assays. To challenge our approach, we focused on the yeast strains belonging to either binary clusters or to the group composed by multiple strains (see Figure 1), i.e., respectively, the C2.A strain for inter-delta cluster 2 and the strains C1.A-D for cluster 1, as indicated in Table 2. SNP selection was performed considering the specific characteristics of the clusters tested, and those having the highest selectivity were prioritized.

### 3.3. Experimental Validation of the Strain-Specific SNPs

**Figure 2:**
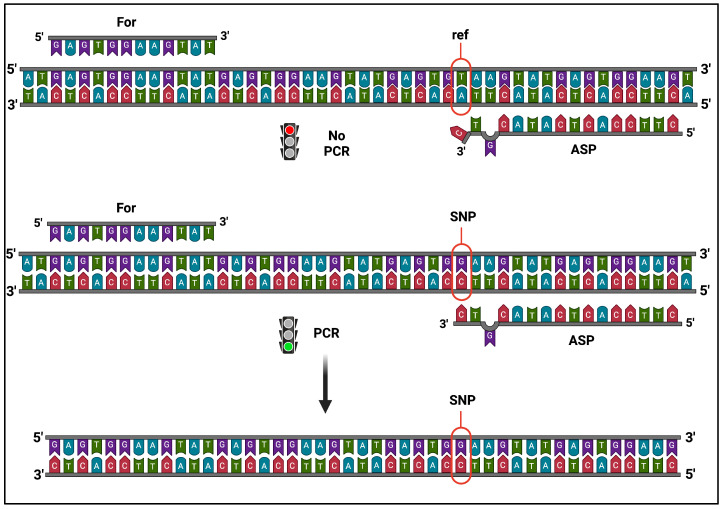
Schematic view of the AS-PCR experimental strategy. PCR amplification by AS primer (ASP) can only occur if the template DNA carries the variant allele (SNP), producing specific DNA fragments, but does not start with the reference sequence (ref). Image was created with BioRender.com.

To confirm the validity of the genetic markers we identified, specific PCR assays were performed using the strategy previously reported [26], with minimal modifications. To perform DNA amplification, PCR reactions required a perfect sequence homology between the template DNA and the nucleotide placed at the 3′-end of the allele-specific (AS) primer. We introduced a second modification in the 3′-region (i.e., a mismatch in the -2 position) to further improve PCR discrimination efficiency, as already suggested [27]. Thus, appropriate AS primer combinations allowed us to amplify specific DNA fragments only in the presence of the proper allele variant in the template, as depicted in Figure 2.

To define the strain-specific SNPs to be investigated, as well as the sequence of AS primers, we manually inspected the list of the SNPs identified by computational analysis for the strains of interest (i.e., C2.A, C1.A-D). We first excluded the SNPs whose sequences were not compatible with the strategy, e.g., where the additional mismatch (position -2) at the 3′-end of the AS primer could not be readily introduced. Then, a final selection was performed by the accurate evaluation of the remaining SNPs, based on the following criteria: (a) the sequence surrounding the SNP locus was analyzed to avoid loci reported as highly polymorphic, as well genomic regions containing either complex or repetitive DNA, and/or judged somehow difficult to be amplified by PCR (e.g., GC-rich); (b) the position of the SNPs in the genome was considered, e.g., preferring SNPs placed on different chromosomes of the same strain, to enhance the assay specificity by monitoring multiple independent loci; (c) the SNPs were also evaluated with respect to the corresponding AS primers, selecting those with comparable features (e.g., Tm), in order to use the same experimental conditions (i.e., temperature of annealing, T_A_) for the PCR reactions, either with single or multiple combinations of primers (multiplex PCR); and (d) SNPs were preferred for the ability to produce DNA fragments with different sizes, therefore readily distinguishable by agarose gel electrophoresis. For the purposes of the present work, yeast strains were tested for either one, two, or three specific SNP genetic markers (Table 2). The selected SNPs were moreover confirmed by Sanger sequencing of the specific genomic loci in the respective strains.

Once the experimental reaction conditions were optimized (e.g., T_A_, see Materials and Methods Section 2), the use of AS primers was expected to allow the DNA amplification of the different strain-specific markers (SNPs), irrespective to their homo- or heterozygosity, distinguishing them from the common allele considered as a reference. The AS-PCR reactions were performed by combining one or more pairs of primers (respectively, uni- and multiplex AS-PCR) to amplify one (or more) of the identified strain-specific variants. The products of the AS-PCR reactions were then subjected to electrophoretic separation in agarose gel, and the DNA fragments observed upon proper staining.

To validate the strategy for the binary group, we firstly started with two SNPs identified for strain A of cluster 2 (C2.A), which were able to distinguish it from the other strain belonging to the same group (C2.B).

**Figure 3:**
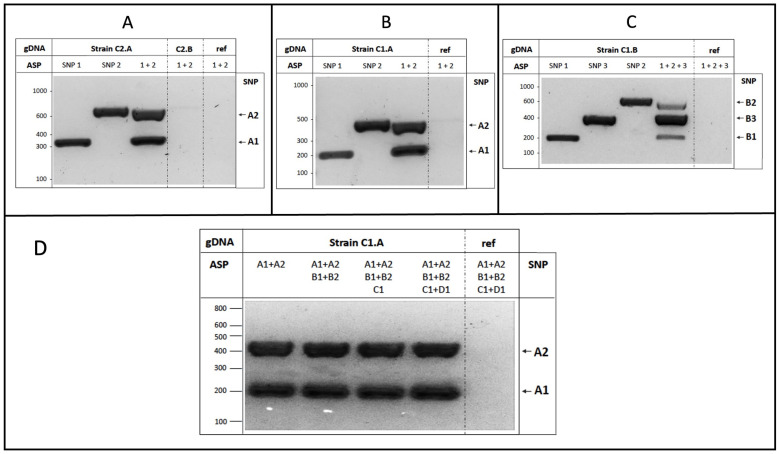
SNP experimental validation. (**A**) Yeast C2.A genomic DNA was used as the template in AS-PCR assays, with primers amplifying either single SNP1 or SNP2, or both variants (1+2). Genomic DNA from either the C2.B strain or S288c (ref) were used as the control; (**B**) C1.A genomic DNA was used as the template in uni- and multiplex AS-PCR assays as indicated, to amplify the two SNP1 and SNP2 strain-specific variants, and their combination; (**C**) the genomic DNA of the C1.B strain was used to detect three different variants (SNP1, SNP2, SNP3) by uni- and multiplex AS-PCR assays, similar to the previous experiments; (**D**) AS-PCR multiplex reactions were performed by using the C1.A genomic template (or S288c as DNA reference, ref) and increasing the number of AS primers, from 4 to 12, without perturbing the specificity and efficiency of the reaction. Generally, after amplification, AS-PCR products were subjected to agarose gel separation and visualized upon staining.

As shown in Figure 3A, the amplification of C2.A genomic DNA only produced the DNA fragments corresponding to the expected size for each locus analyzed, without the presence of unspecific electrophoretic bands. The specificity of the reactions was further corroborated by the absence of amplification using as a template the genomic DNA of either the C2.B strain or the S288c reference carrying the common variant shared by all strains considered in the bioinformatic dataset. The AS-PCR equally amplified both identified variants (SNP1 and SNP2), with similar results in the case of the uniplex PCR (i.e., single SNP, 2 primers) and the multiplex reaction (SNP1 + SNP2, 4 primers). Overall, the experimental evidence demonstrates that the SNPs can be specifically recognized through carefully optimized PCR reactions by using appropriate combinations of AS primers.

We then focused on the SNPs identified for the strains belonging to cluster 1. We started with the C1.A strain, confirming that both SNP1 and SNP2 were able to discriminate the strain, as indicated by the presence of DNA amplification fragments in uni- and multiplex AS-PCRs only in the presence of C1.A genomic DNA as the template (Figure 3B).

For the yeast C1.B strain, we further optimized the assay in order to recognize more than two SNPs on the same genome by the set-up of the multiplex AS-PCR containing six primers. As shown in Figure 3C, each C1.B-specific SNP was readily detected, but displayed quantitative (but not qualitative) differences in the multiplex AS-PCR amplification. We then extended the experimental analysis to the C and D strains of cluster 1, selecting one specific SNP characterizing each strain (C1.C and C1.D, as indicated in Table 2), which were both experimentally validated and used in the assays described below.

Importantly, to increase the number of strains/SNPs that could be simultaneously distinguished by the multiplex AS-PCR, we performed a series of reactions by increasing the number of AS primers added in the same tube (maintaining identical reaction conditions). As shown in Figure 3D, no effects on the specificity or intensity of the amplicons (for both SNP1 and SNP2 of the C1.A strain) were observed in the presence of additional AS primers, up to twelve total oligonucleotides.

Collectively, these data show that the SNPs identified here can be considered as highly performing strain-specific markers, indicating that multiple SNPs can be simultaneously detected by single AS-PCR reactions.

We indeed checked the use of SNPs as markers by generating binary combinations of pure strains, which mirrored commercial blends of winery yeasts (as available from suppliers). Cells from each C1.A and C2.A strain were propagated in liquid medium, properly mixed (1:1), diluted, plated, and then grown for two days on solid medium. When single colonies formed, they displayed very a similar morphology, as expected. Some randomly selected colonies were processed and subjected to multiplex AS-PCR analysis, i.e., containing the AS primers for the amplification of the two SNP markers carried by each strain (4 SNPs, 8 primers).

**Figure 4:**
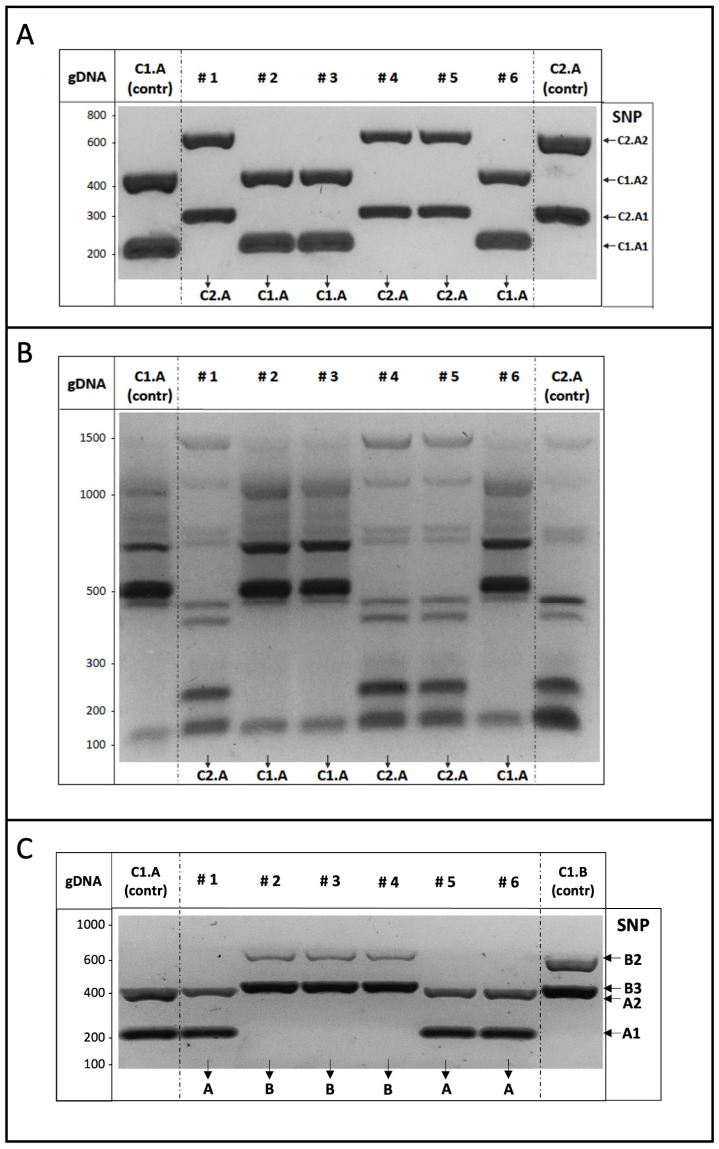
(**A**) Binary mixture genotyping by multiplex ASP-PCR assay. From a 1:1 mixture of C1.A and C2.A strains, six yeast colonies (#1–6) were randomly selected, and genomic DNA was used as a template in the multiplex AS-PCR assay to detect two SNPs for each strain. Colony identity can be determined by banding comparison with the parental C1.A (left) and C2.A (right) strains, considered as the reference (contr). (**B**) Yeast genotyping. As in Figure 1, genomic DNA purified from the indicated yeast colonies was used as the template for inter-delta PCR assays to compare the profiles of the colonies and the parental (C1.A and C2.A) strains. (**C**) Binary mixture genotyping by multiplex ASP-PCR assay. Strains C1.A and C1.B were mixed (1:1) and genomic DNA from six independent colonies was used as the template in the multiplex AS-PCR assay to amplify two SNPs for each component strain. Colony identity was assigned by a profile comparison with the two C1.A and C1.B parental strains, considered as the reference (contr).

The experimental results obtained from the analysis of six independent colonies selected from the (1:1) mixture of C1.A and C2.A strains are shown in Figure 4A. Compared to the parental C.1A and C2.A strains, considered as controls, the DNA fragments amplified by the genomic DNA of each colony specifically corresponded to those of one or the other of the two yeast strains comprising the mixture, revealing their identity. Furthermore, as the C1.A and C2.A strains can be distinguished by the inter-delta profiles, genomic DNA of the same colonies was subjected to such genotyping analysis, which independently confirmed the identity of the yeast colonies (Figure 4B).

Identical results were further obtained by a similar multiplex AS-PCR analysis, performed on the yeast colonies selected from the binary mixture between the A and B strains of the same cluster (1) (Figure 4C). Also in this experiment, the multiplex AS-PCR assay confirmed that SNPs can be used as genetic markers to recognize yeast strains with very similar genomic configurations, indicating major discriminative ability with respect to inter-delta genotyping.

Thereafter, we experimentally addressed the efficiency of the multiplex AS-PCR with twelve primers to specifically and simultaneously recognize six different SNPs belonging to the four strains of cluster 1 (C1.A-D, Table 2).

**Figure 5:**
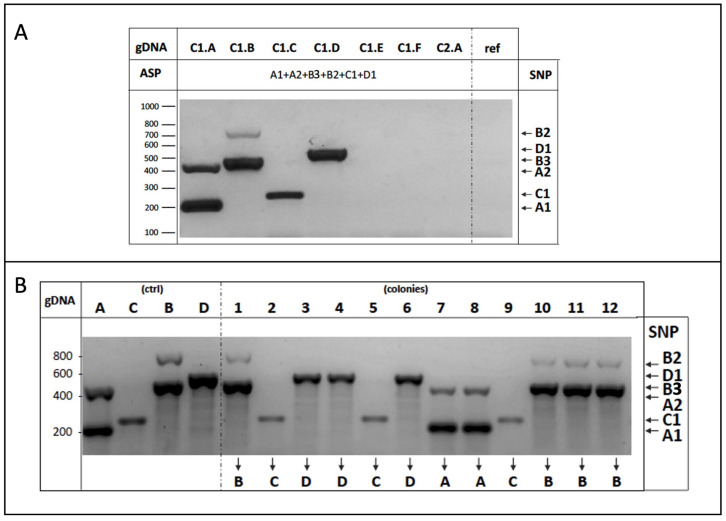
(**A**) Multiplex AS-PCR experimental validation. Yeast genomic DNA purified from the indicated strains of cluster 1 were used as a template in the multiplex AS-PCR assay to detect the six different SNPs indicated on the right. No amplification occurred by using as a template the DNA from either the W303 laboratory strain (ref), other strains of the same C1 cluster (C1.E, C1.F), or those belonging to another group (C2.A); (**B**) Genotyping of multiple strain mixture by multiplex ASP-PCR assay. Equal amounts of yeast cells of cluster 1 strains (A,B,C,D) were mixed, and genomic DNA from twelve single colonies (1–12) were used as a template in the multiplex ASP-PCR assay to amplify the SNPs indicated on the right. Colony identity was assigned by profile comparison with the four parental strains (C1.A-D), considered as the reference, on the left (contr).

As shown in Figure 5A, the experimental results indicate the presence of PCR products of the expected size according to the strain’s genomic DNA used as a template in the reaction. Importantly, no unspecific amplification occurred for the DNA obtained from either strictly related strains (as C1.E-F belonging to the same cluster (1)), from another group (i.e., C2.A strain), or from the laboratory W303 strain (ref), as it was expected since all these strains share an identical sequence in the corresponding locus.

We finally generated a multiple mixture composed of four yeast strains sharing identical inter-delta profiles by equally mixing cells of the C1.A, B, C, and D strains, as described above. Twelve independent colonies were randomly selected, processed, and multiplex AS-PCR reactions (six SNPs, twelve primers) were performed using the genomic DNA of each colony as a template. Resulting data (Figure 5B) demonstrate that a single multiplex AS-PCR reaction is able to determine the identity of each yeast colony analyzed, i.e., to specifically identify the yeast strains, definitively supporting the strategy reported here.

### 3.4. Application of the ASP-PCR Strategy in Laboratory Assays

The AS-PCR strategy was then challenged in the characterization of some yeast strains during a fermentation assay in stressing conditions. Notably, a major problem in winemaking is stuck fermentation, where yeast cells prematurely undergo quiescence and stop metabolic activity [37]. Compared to normal conditions, lower levels of ethanol are produced and higher amounts of sugars (mainly fructose) remain to be consumed, indicating that must fermentation is incomplete. Very often, to restart and conclude the process, specific yeast strains are added, which are able to either consume residual sugars, survive in an uncomfortable environment (high ethanol, copper), or tolerate biotic stress, e.g., the competition with different yeast strains.

In the laboratory, we mimicked stuck fermentation conditions as described in Section 2 (Materials and Methods Section). Commercial white wine (ethanol 10.9% *v*/*v*) was added to the amount of fructose required to support the additional production of ethanol (1.5% *v*/*v*) upon fermentation. Two independent bottles containing this substrate (yeast-free) were inoculated with the same number of yeast cells (5 × 10^6^ cells/mL) without preliminary acclimatization to increase biotic stress. Cells were collected from a mixture of eleven different yeast strains (namely, C1.A-D, C2.A, C3.A, U_4_, U_10_, U_15_, U_16_, U_17_), previously assembled by mixing equal amounts of each strain (4.5 × 10^5^ cells/mL). Fermentation and ethanol production were constantly monitored for 22 days, when ethanol levels were stabilized.

**Figure 6:**
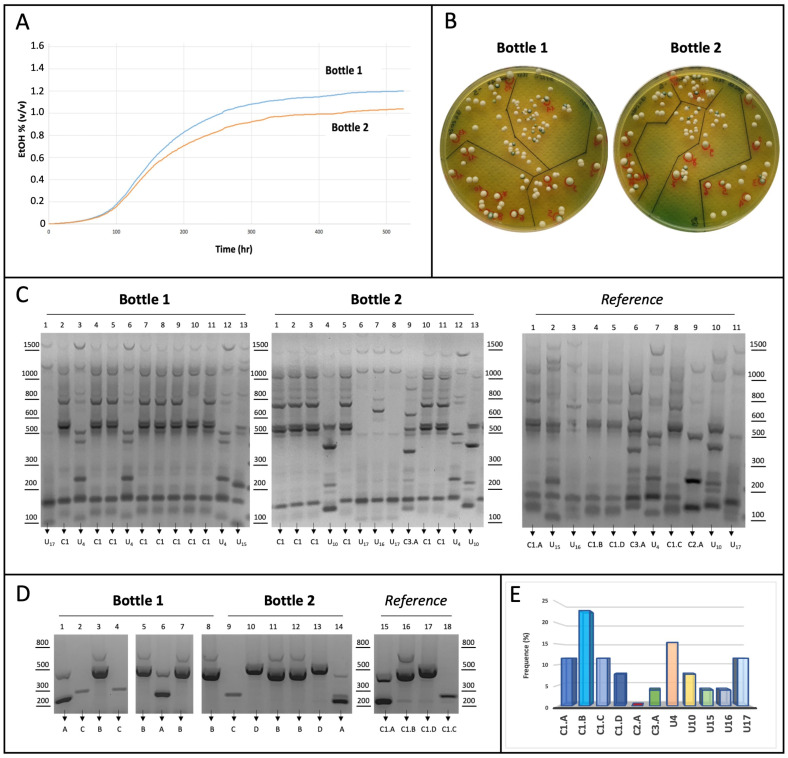
(**A**) Fermentation kinetics. Two independent bottles (1 and 2) containing white wine added to 25 g/L of fructose were inoculated with a mixture of eleven yeast strains leading to substrate fermentation. Ethanol production was constantly monitored for 22 days and finally represented as kinetic curves for each bottle. (**B**) Colony isolation. WL nutrient agar plates were used to isolate *S. cerevisiae* cells by spreading selected dilutions from bottles 1 and 2. After incubation for 48 h at 28 °C, thirteen colonies were randomly selected from a plate of each bottle. (**C**) Genotyping by inter-delta analysis. Genomic DNA purified from each indicated yeast colony was used as a template in the inter-delta PCR assays. Each colony (lanes 1–13) was isolated from bottles 1 (left) and 2 (middle); gDNA from pure strains was used as a reference for pattern comparison (right). DNA products were subjected to agarose gel separation and visualized upon staining. (**D**) Genotyping by multiplex AS-PCR assay. Genomic DNA from the fourteen colonies (1–14) assigned to cluster 1 were used as a template in the multiplex AS-PCR assay to amplify the strain-specific SNPs (as in Figure 5B). Colony identity was assigned by profile comparison with the four reference strains on the right. (**E**) Distribution frequence (%) of the yeast strains during the stress test. Data from inter-delta and multiplex AS-PCR assays were combined to establish the frequence of yeast strains upon colony genotyping.

As indicated by the kinetics shown in Figure 6A, the fermentative process (although slow) led to the final production of ethanol in both bottles (1.0–1.2% *v*/*v*). After fermentation, the *S. cerevisiae* strains were isolated from each bottle on WL agar plates, and twenty-six independent colonies (thirteen from each bottle) were selected. The genomic DNA extracted from each colony was used for a first genotyping by inter-delta PCR assay (Figure 6C), which allowed us to recognize unique-patterned strains, but not to identify those belonging to cluster 1, which were overall the most frequent (54%). Therefore, a multiplex AS-PCR was performed for the fourteen clones belonging to cluster 1. As shown in Figure 6D, the multiplex AS-PCR assay led to strain-level identification for all colonies. Therefore, the data collectively support the major discriminative ability of the multiplex AS-PCR assay compared to the inter-delta analysis. Despite the limited number of colonies tested, the results of the competition assay indicate that the strains of cluster 1 seem more capable of coping with biotic stress, and among them, the C1.B strain appears to be the most capable, as it appeared more frequently at the end of the test (Figure 6E). Instead, the C2.A strain could be mostly impaired by such experimental conditions, since no colonies have been detected in our analysis.

## 4. Discussion

The environmental microbiome is a natural “gold mine” to identify microorganisms suitable to multiple biotechnology applications. Yeasts are of special interest to support fermentative processes, and winemaking industries are continuously promoting the isolation of new *S. cerevisiae* strains endowed with peculiar phenotypes, which could confer interesting properties to the wine bouquet. Nevertheless, the commercial use of newly isolated yeast strains also requires the development of specific techniques to trace them unequivocally and to perform the quality control of the final products. Importantly, yeast strains for oenological purposes (e.g., fermentation starters) are commercially provided as “active dry yeast” (ADY) products, often composed of mixtures of two or more (pure) yeast strains. As mentioned, multiple analysis by sequence-specific PCR assays can be performed to identify yeast species, e.g., by the investigation of ribosomal genes, and genome fingerprinting protocols have been developed to discriminate yeast cells at the strain level [10]. However, most methods may be limited by either the high variability of the experimental data or the critical requirement of time and materials also related to specific instruments’ availability. Overall, the inter-delta analysis is today the most discriminating (and cost-effective) PCR-based assay among various fingerprinting methods [21]. Many commercial *S. cerevisiae* strains can be identified by this approach, revealing distinct amplicon patterns for different strains. However, yeast strains carrying very similar genome configurations cannot be identified by inter-delta analysis, as they may produce identical patterns despite being endowed with distinct phenotypic characteristics [20,22]. In this work, we established a new method to overcome such limitations of the inter-delta assay, maintaining, however, the efficiency, speed, and low cost of the PCR-based approaches. As a starting point, we considered that most differences between the genomes of organisms belonging to the same species (as *S. cerevisiae* strains) consist of either single nucleotide polymorphisms (SNPs) or small insertions/deletions, which can be precisely identified by the comparation of multiple genomes through computational analysis. We further considered that such SNPs may be readily discriminated by PCR assays using allele-specific (AS) primers, which are designed to only amplify the selected nucleotide variants. To test our strategy, we investigated a database containing the genomes of about 30 different *S. cerevisiae* strains belonging to the private collection of the partner company and functionally characterized, some of which shared similar genome configurations, resulting in common inter-delta profiles, despite being endowed with peculiar properties. Interestingly, a list of strain-specific SNPs resulted from the bioinformatic analysis of the NGS data composing the yeast database, and upon selection, some variants were further investigated by a series of experimental assays. Overall, the data demonstrate that the SNPs can be specifically and efficiently recognized through carefully optimized PCR reactions by using appropriate combinations of AS primers. We also provide evidence as *proof of principle* that a single SNP can be sufficient to identify one strain among (at least) the other strains included in the database used for computational analysis. The data further indicated that the multiplex PCR can be implemented to simultaneously identify up to six strains in the same assay, and possibly, the number of primers (and strains) can be increased further. The data moreover support the idea that the SNPs identified can be considered as highly performing strain-specific markers, since the stability of the SNPs over time was further confirmed by performing AS-PCR analysis on yeast strains produced in different years and by different producers, observing identical results of PCR amplifications. Importantly, the multiplex AS-PCR strategy (targeting six strain-specific SNPs) allowed us to unambiguously discriminate four closely related yeast strains, sharing common inter-delta patterns, as demonstrated by the similar results obtained in both experimental conditions we tested, i.e., balanced artificial mixtures of the four strains, and during the competitive stress test with multiple yeast strains.

Unlike other available procedures, possibly discriminating yeast strains sharing common inter-delta profiles (e.g., microsatellite loci), the strategy proposed here could represent the best molecular tool to trace a strain of interest by an optimized PCR assay, maintaining a low cost and short time for *S. cerevisiae* genotyping. It is of special interest when the analysis is systematically performed, as in the quality control step of commercial yeast production at the industrial scale.

Whole genome sequencing is however required to apply our strategy to a defined yeast strain, but considering the relatively small genome of *S. cerevisiae* (13 Mbp), the complete sequencing by the NGS technique of specific strains can be performed at a relatively low cost, while providing the most crucial piece of information about that yeast strain. Paradoxically, it would be expected that by increasing the number of sequenced strains in the collection’s database, some variants will likely appear in the new genomes and will therefore no longer be strain-specific. Consistently, the identification of strain-specific SNPs could become increasingly challenging when strains are closely related, possibly leading to design efficient primers for strain groups, but no primer combinations that identify multiple groups. Furthermore, selecting appropriate primers for multiplex PCR could become particularly challenging when analyzing mixtures containing numerous strains, but our approach allowed us to successfully identify four strains mixed together, or with additional strains, which were initially inoculated in similar concentrations. However, when strains are present in unequal proportions within a mixture, more PCR tests could be required to detect those present in a lower abundance, and to analyze mixtures with highly unbalanced strain proportions, real-time PCR could be employed to precisely quantify the amplicon concentrations.

## 5. Conclusions

In this study, we aimed to overcome the limitations of inter-delta analysis in identifying *S. cerevisiae* strains with highly similar genetic characteristics. Our approach relies on the availability of next-generation sequencing (NGS) data for the strains under investigation, which exhibit identical inter-delta profiles, in order to detect single nucleotide polymorphisms (SNPs) that can differentiate them. The genetic differences identified through computational analysis enable the design of specific primers for the unique detection of these SNPs and, consequently, of the strain itself. This strategy allows for the continued use of a fast and cost-effective PCR-based methods for screening or quality control when genome sequencing the relevant strain(s).

## Figures and Tables

**Table 1:** Number of SNPs identified by the computational analysis of all twenty-eight strains (total and unique). Suitable variants were selected considering either homo- or heterozygous SNPs, whereas small insertions/deletions (InDels) were excluded. Upon quality assessment filtering, unique strain-specific variants have been identified, ranging from 70 to >3000 within the entire dataset.

#	Strain Name	SNP (Total)	SNP (Unique)
1	C1.A	68,678	82
2	C1.B	69,587	71
3	C1.C	69,297	98
4	C1.D	70,513	90
5	C1.E	68,185	205
6	C1.F	69,748	106
7	C2.A	65,336	314
8	C2.B	65,563	377
9	C3.A	64,667	113
10	C3.B	60,521	192
11	C4.A	54,285	135
12	C4.B	54,552	117
13	C5.A	58,957	1449
14	C5.B	57,608	149
15	U1	54,795	1981
16	U2	58,235	784
17	U3	57,917	475
18	U4	54,567	2299
19	U5	59,393	1653
20	U6	56,098	1994
21	U7	66,656	1085
22	U8	57,025	2743
23	U9	66,642	654
24	U10	61,974	3428
25	U11	54,188	1376
26	U12	54,640	3123
27	U13	63,597	2514
28	U14	62,550	2127

**Table 2:** Single nucleotide polymorphisms experimentally validated. For all nine variants (SNPs) investigated in this study, the chromosomal position in the yeast genome and the size of the PCR product are indicated for each strain. The AS primer sequences are in Supplementary Table S1.

Strain	SNP	Locus Chr–Position		PCR Product Size (DNA SNP) (bp)
C1.A	1	IV	369,222	203
C1.A	2	XIII	796,963	430
C1.B	1	I	194,376	223
C1.B	2	XV	803,410	689
C1.B	3	IV	1,508,363	451
C1.C	1	XV	357,173	244
C1.D	1	II	289,545	505
C2.A	1	VI	83,415	289
C2.A	2	VII	854,569	599

## Data Availability

The original contributions presented in the study are included in the article, further inquiries can be directed to the corresponding author.

## Supplementary Materials

The following supporting information can be downloaded at: https://www.mdpi.com/article/10.3390/foods14132379/s1, Table S1: SNP primer sequences used in this work; Table S2: List of strain-specific SNPs (total); Supplementary Figure S1: Copper sensitivity of yeast strains; Supplementary Materials: Yeast strain descriptions; additional procedure details.

## References

[1] Goddard M.R. (2008). Quantifying the complexities of *Saccharomyces cerevisiae*’s ecosystem engineering via fermentation. Ecology.

[2] Salvadó Z., Arroyo-López F.N., Barrio E., Querol A., Guillamón J.M. (2011). Quantifying the individual effects of ethanol and temperature on the fitness advantage of *Saccharomyces cerevisiae*. Food Microbiol..

[3] Lopes C.A., Rodríguez M.E., Sangorrín M., Querol A., Caballero A.C. (2007). Patagonian wines: The selection of an indigenous yeast starter. J. Ind. Microbiol. Biotechnol..

[4] Marsit S., Dequin S. (2015). Diversity and adaptive evolution of *Saccharomyces* wine yeast: A review. FEMS Yeast Res..

[5] Rainieri S., Pretorius I.S. (2000). Selection and improvement of wine yeasts. Ann. Microbiol..

[6] Mateo J.J., Jimenez M., Pastor A., Huerta T. (2001). Yeast starter cultures affecting wine fermentation and volatiles. Food Res. Int..

[7] Nykänen L. (1986). Formation and Occurrence of Flavor Compounds in Wine and Distilled Alcoholic Beverages. Am. J. Enol. Vitic..

[8] Romano P., Capece A., Serafino V., Romaniello R., Poeta C. (2008). Biodiversity of wild strains of *Saccharomyces cerevisiae* as tool to complement and optimize wine quality. World J. Microbiol. Biotechnol..

[9] Kurtzman C., Fell J.W., Boekhout T. (2011). The Yeasts: A Taxonomic Study.

[10] Guillamón J.M., Barrio E. (2017). Genetic Polymorphism in Wine Yeasts: Mechanisms and Methods for Its Detection. Front. Microbiol..

[11] Bezerra-Bussoli C., Alves Baffi M., Gomes E., Da Silva R. (2013). Yeast Diversity Isolated from Grape Musts During Spontaneous Fermentation from a Brazilian Winery. Curr. Microbiol..

[12] Díaz C., Molina A.M., Nähring J., Fischer R. (2013). Characterization and dynamic behavior of wild yeast during spontaneous wine fermentation in steel tanks and amphorae. BioMed Res. Int..

[13] Arias C., Burns J.K., Friedrich L.M., Goodrich R.M., Parish M.E. (2002). Yeast species associated with orange juice: Evaluation of different identification methods. Appl. Environ. Microbiol..

[14] Esteve-Zarzoso B., Belloch C., Uruburu F., Querol A. (1999). Identification of yeasts by RFLP analysis of the 5.8S rRNA gene and the two ribosomal internal transcribed spacers. Int. J. Syst. Bacteriol..

[15] Muyzer G. (1999). DGGE/TGGE a method for identifying genes from natural ecosystems. Curr. Opin. Microbiol..

[16] Ivey M.L., Phister T.G. (2011). Detection and identification of microorganisms in wine: A review of molecular techniques. J. Ind. Microbiol. Biotechnol..

[17] Pérez-Martín F., Seseña S., Fernández-González M., Arévalo M., Palop M.L. (2014). Microbial communities in air and wine of a winery at two consecutive vintages. Int. J. Food Microbiol..

[18] Esteve-Zarzoso B., Fernández-Espinar M.T., Querol A. (2004). Authentication and identification of *Saccharomyces cerevisiae* ‘flor’ yeast races involved in sherry ageing. Antonie Van Leeuwenhoek.

[19] Vaudano E., Garcia-Moruno E. (2008). Discrimination of *Saccharomyces cerevisiae* wine strains using microsatellite multiplex PCR and band pattern analysis. Food Microbiol..

[20] Legras J.L., Karst F. (2003). Optimisation of interdelta analysis for *Saccharomyces cerevisiae* strain characterisation. FEMS Microbiol. Lett..

[21] Siesto G., Capece A., Sipiczki M., Csoma H., Romano P. (2013). Polymorphism detection among wild *Saccharomyces cerevisiae* strains of different wine origin. Ann. Microbiol..

[22] Pfliegler W.P., Sipiczki M. (2016). Does fingerprinting truly represent the diversity of wine yeasts? A case study with interdelta genotyping of *Saccharomyces cerevisiae* strains. Lett. Appl. Microbiol..

[23] Cottrell M.T. (2022). Fingerprinting *Saccharomyces cerevisiae* Strains Using Next Generation Sequencing of PCR Amplicons Generated from Delta Elements. J. Am. Soc. Brew. Chem..

[24] Amberg D.C., Burke D.J., Strathern J.N. (2005). Methods in Yeast Genetics: A Cold Spring Harbor Laboratory Course Manual.

[25] Bray M.A., Vokes M.S., Carpenter A.E. (2015). Using Cellprofiler for automatic identification and measurement of biological objects in images. Curr. Protoc. Mol. Biol..

[26] Ye S., Dhillon S., Ke X., Collins A.R., Day I.N. (2001). An efficient procedure for genotyping single nucleotide polymorphisms. Nucleic Acids Res..

[27] Medrano R.F., de Oliveira C.A. (2014). Guidelines for the tetra-primer ARMS-PCR technique development. Mol. Biotechnol..

[28] Korbie D.J., Mattick J.S. (2008). Touchdown PCR for increased specificity and sensitivity in PCR amplification. Nat. Protoc..

[29] Basile A., De Pascale F., Bianca F., Rossi A., Frizzarin M., De Bernardini N., Bosaro M., Baldisseri A., Antoniali P., Lopreiato R. (2021). Large-scale sequencing and comparative analysis of oenological *Saccharomyces cerevisiae* strains supported by nanopore refinement of key genomes. Food Microbiol..

[30] DePristo M.A., Banks E., Poplin R., Garimella K.V., Maguire J.R., Hartl C., Philippakis A.A., del Angel G., Rivas M.A., Hanna M. (2011). A framework for variation discovery and genotyping using next-generation DNA sequencing data. Nat. Genet..

[31] Li H., Durbin R. (2009). Fast and accurate short read alignment with Burrows–Wheeler transform. Bioinformatics.

[32] Peter J., De Chiara M., Friedrich A., Yue J.X., Pflieger D., Bergström A., Sigwalt A., Barre B., Freel K., Llored A. (2018). Genome evolution across 1,011 *Saccharomyces cerevisiae* isolates. Nature.

[33] Felsenstein J. PHYLIP (Phylogeny Inference Package) Version 3.6. Distributed by Author, 2005. Department of Genome Sciences, University of Washington, Seattle. https://phylipweb.github.io/phylip/.

[34] Xufre A., Albergaria H., Gírio F., Spencer-Martins I. (2011). Use of interdelta polymorphisms of *Saccharomyces cerevisiae* strains to monitor population evolution during wine fermentation. J. Ind. Microbiol. Biotechnol..

[35] Castillo M.M., Parra N., Câmara J.S., Khadem M. (2025). Unveiling the Regional Identity of Madeira Wine: Insights from *Saccharomyces cerevisiae* Strains Using Interdelta Analysis. Beverages.

[36] Liti G., Carter D.M., Moses A.M., Warringer J., Parts L., James S.A., Davey R.P., Roberts I.N., Burt A., Koufopanou V. (2009). Population genomics of domestic and wild yeasts. Nature.

[37] Bisson L.F. (1999). Stuck and sluggish fermentations. Am. J. Enol. Vitic..

