# Supplementary material for: A Novel PCR-Based Tool to Trace Oenological Saccharomyces cerevisiae Yeast by Monitoring Strain-Specific Nucleotide Polymorphisms
Source: Foods. 2025 Jul 4;14(13):2379. doi: 10.3390/foods14132379 (PMC12248937; doi:10.3390/foods14132379)
Supplement: Supplementary file 1 [file foods-14-02379-s001.zip › Baldisseri et al, Supplementary Materials (revised).pdf]

“A novel PCR-based tool to trace oenological *Saccharomyces cerevisiae* yeast by monitoring strain-specific nucleotide polymorphisms” by Baldisseri *et al*

Supplementary Material.

A) Relevant features of some yeast strains used in this study are indicated:

Strain	DBVPG #ID	Geographical origin	Main application	Commercial name	Producer
C1.A	DBVPG 64 SF	Italy (Piemonte)	Barrel-aged red wine (*Nebbiolo*)	*Enartisferm Vintage Red*	*Enartis*
C1.B	-	France (Reims)	White wine (*Chardonnay*)	*Uvaferm DV10*	*Lallemand*
C1.C	DBVPG 41 SF	Italy (Piemonte)	Red wine (*Pinot noir*)	*Enartisferm Q-Grace*	*Enartis*
C2.A	DBVPG 78 SF	Italy (Campania)	White wine (dry)	*RE515*	*Ever*
C2.B	DBVPG 62 SF	France (Val du Rhone)	Red wine (*Syrah*)	*Enartisferm Q5*	*Enartis*
C3.A	-	-	White wine (*Sauvignon*)	*Zymaflore X5*	*Laffort*
C3.B	DBVPG 76 SF	Italy (Lazio)	White wine (*Sauvignon*)	*Quarzo*	*Ever*
C5.B	DBVPG 77 SF	Italy (Friuli-VG)	White wine (dry)	*Mycoferm Pronature*	*Ever*
U3	DBVPG 72 SF	California (Napa Valley)	Aged red wine	Enartisferm D20	*Enartis*
U4	DBVPG 52 SF	Italy (Emilia Romagna)	White wine (dry)	*Enartisferm Q Citrus*	*Enartis*
U9	DBVPG 73 SF	Italy (Toscana)	Aged red wine	*Enartiferm SPLZ07*	*Enartis*

B) Primer design procedure details

The procedure to design the primers used in this study was based on the PRIMER1 software, setting the parameters as in the following:

The chromosomal sequence (2000 bp) surrounding the considered SNP was inserted into the database, establishing the reverse primer, consisting of 27 bp and whose 3' end was located on the SNP nucleotide. Then, software parameters were exactly defined:

- T_m value: min 58.0 - max 62.0 °C (maximal T_m difference: ± 2°C)
- Reference genome for primer Pair Specificity Checking Parameters: *Saccharomyces cerevisiae* S288C strain (taxID:559292)
- Primer specificity stringency: Primer must have at least **4** total mismatches to unintended targets, including at least 3 mismatches within the last 6 bps at the 3' end. Targets with 6 or more mismatches to the primer were discarded
- Target amplicon maximal size: 1000 bp
- Primer GC content (%): min 40.0 - max 70.0
- Maximal Self Complementarity: any=6.00
- Maximal Pair Complementarity: any=6.00

The primers provided by the system have been filtered by evaluating that: 1) primers pairs (e.g. For and Rev) had similar Tm and GC content (%); 2) primers had the lowest Self Complementarity and Self 3’ Complementarity values; 3) amplicon length differed of (at least) 200 bp, in order to distinguish by agarose gel electrophoresis the PCR products of multiplex assays.

Once the suitable primer pair has been identified, the second mismatch was added to the Reverse primer, replacing the third-to-last base. Primers were finally checked using MFEprimer software.

If some of the parameters did not meet these requirements, another SNP identified by sequencing has been evaluated with identical procedure.

Supplementary Figure S1. *Copper sensitivity assay.*
Viability assay was performed for the indicated yeast strains used in this study. Exponentially growing *S. cerevisiae* cells of strains were normalized to OD_{600}=1, serially diluted (1:10), and spotted on standard medium (YPD) supplemented with the indicated concentrations of $CuSO_4$. Plates were incubated at 28 °C for 3 days.

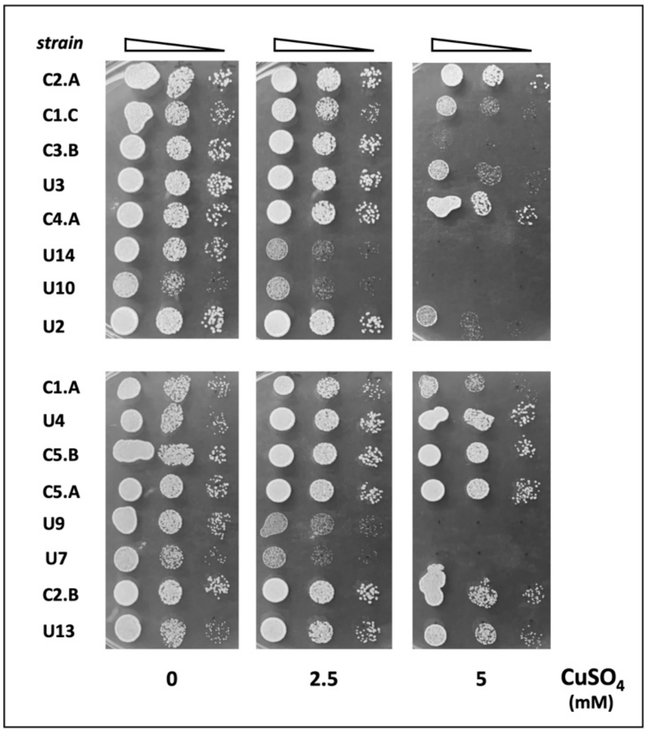

Supplementary Table ST1. Allele-specific (AS) primer pairs for the five *S. cerevisiae* strains analyzed. The added mismatch at position -2 from the 3’–end of the allele-specific primer is underlined and the nucleotide corresponding to the SNP position is in bold. The allele-specific primer could be either the Forward or the Reverse oligo.

Strain	SNP	Forward primer (5’ 3’)	Reverse primer (5’ 3’)	Annealing Temperature (°C)
C1.A	A1	GTAACCATGAAGTGTAAGGCAA**A**	AGCGGACTTGACTAGACTTATTAC	57.5
C1.A	A2	CCTTAGCGATTTCAAGTGCAAA**T**	GATTTCTTAGAATATTATCCCTCTCTGC	58.0
C1.B	B1	GTTACTCTGAGGACGATTCTGAT	ATAGAAGTGTTGACGGTGGT**T**	57.5
C1.B	B2	CCTTTTCCAAAGTAGCGTGTATC	GTTTGTTAAGTTAGTGGCCACT**C**	58.0
C1.B	B3	GTATACGTGTTCTAGCATACAAGTTAG	GCGCATGTAGAAATAACGGTA**A**	57.5
C2.A	C2.A1	GGGAACAGATGTCTTGACGA**T**	AGTCTCAGAAACAAAAGTTCTTTCC	58.0
C2.A	C2.A2	GTTAAGTACTGGATGCAGATCTTTT	GCAAGGAAAGTACAGGTAAAAT**T**	57.0
C1.C	C1	GGGAAGGGGTTCACGTC**T**	TGGAAAACAAAGCGTAGTTTCAC	58.5
C1.D	D1	GAGGCGCATATCGAAGCG**A**	ACATCCGGTGTATCTCTTGCAG	60.0
